# Effectiveness and safety of cefotaxime combined with avibactam for treating multidrug-resistant *E coli* infections: A systematic review and meta-analysis

**DOI:** 10.1097/MD.0000000000036938

**Published:** 2024-01-19

**Authors:** Geming Liu, Jia Qiu, Yang Liu, Zhisen Liu

**Affiliations:** a Department of Pharmacy, Affiliated Hospital of Jilin Medical University, Jilin, Jilin Province, China; b Department of Pharmacy, Affiliated Hospital of Beihua University, Jilin, Jilin Province, China; c Department of Pharmacy, Jilin Chemical Hospital, Jilin, Jilin Province, China; d Clinical Pharmacy, Affiliated Hospital of Jilin Medical University, Jilin, Jilin Province, China.

**Keywords:** avibactam, cefotaxime, meta-analysis, multidrug-resistant *E coli*

## Abstract

**Background:**

Multidrug-resistant *Escherichia coli* infections are a global health challenge, notably in North America, Europe, Asia, and Africa. This systematic review and meta-analysis evaluates the effectiveness and safety of cefotaxime combined with avibactam, aiming to mitigate these infections’ impact and lessen their burden on healthcare systems worldwide.

**Methods:**

Following the Preferred Reporting Items for Systematic Reviews and Meta-Analyses and PICO frameworks, we conducted a comprehensive literature search across 4 primary databases on May 6, 2023. Studies evaluating the efficacy and safety of cefotaxime and avibactam were included. Key outcomes included treatment success, adverse effects, and microbiological eradication. Quality assessment utilized the Cochrane Collaboration Risk of Bias instrument. Heterogeneity was analyzed using chi-square statistics and the I^2^ index. Both fixed- and random-effects models were applied as appropriate. Publication bias was rigorously evaluated using Egger linear regression test and funnel plot analysis, ensuring the study’s integrity and reliability.

**Results:**

The clinical cure rate derived from 8 studies showed no significant difference between the treatment groups (odds ratio [OR] = 1.97, 95% CI: 0.69 to 1.36, *P* = .86). Analysis of the bacterial clearance rate from the 5 studies also indicated no significant difference (OR = 0.97, 95% CI: 0.42 to 2.25, *P* = .36). Notably, a reduced mortality rate favoring the experimental group was observed in 6 studies (OR = 0.64, 95% CI: 0.44 to 0.92, *P* = .012). Comprehensive sensitivity analyses and the assessment of publication bias strengthened the reliability of the results.

**Conclusions:**

Ceftazidime combined with avibactam significantly reduced mortality among patients with multidrug-resistant *Escherichia coli* infections, indicating its potential as a therapeutic option, especially for carbapenem-resistant Enterobacteriaceae. However, extensive large-scale clinical trials are required to validate these findings.

## 1. Introduction

The emergence and rapid escalation of antimicrobial resistance represents one of the most daunting challenges facing modern healthcare systems globally. Notably, multidrug resistant Enterobacteriaceae (MDREB) have been a focal point of concern because of their capacity to withstand an array of antibiotics, thereby posing a serious threat to patient health and public safety. Traditionally, carbapenem antibiotics have been the cornerstone for treating severe infections caused by MDREB.^[1,2]^ However, the clinical landscape has been considerably complicated by the emergence of carbapenem-resistant Enterobacteriaceae (CRE).

The upward trajectory in the frequency of CRE detection is not only alarming, but also indicative of a deepening crisis in antimicrobial resistance. As conventional therapeutic agents, including carbapenems, become increasingly ineffective, healthcare practitioners are compelled to seek alternative treatment paradigms. The inadequacy of the existing therapeutic arsenal against CRE necessitates accelerated drug discovery and development initiatives.^[3,4]^ In this context, an innovative combination of cefotaxime and avibactam (CAZ/AVI) has garnered significant attention. Representing a novel class of β-lactam/β-lactamase inhibitor combinations, CAZ/AVI promises to fill a significant gap in our therapeutic toolkit. Preliminary studies have shown that this combination effectively targets a wide array of enzymes responsible for β-lactam resistance, thereby restoring the antimicrobial activity of β-lactam components. Furthermore, in vitro experiments and animal models have demonstrated the efficacy of CAZ/AVI against a myriad of MDREB pathogens, including CRE strains.^[5,6]^ It is worth noting that the action of the combination is not confined to combating CRE alone but extends to treating infections caused by other multidrug-resistant organisms as well.

Nonetheless, the rapid deployment of CAZ/AVI in clinical settings has raised questions regarding its long-term effectiveness and safety profile. Although preliminary clinical trials have indicated the potential benefits of CAZ/AVI, comprehensive data regarding its safety and efficacy, particularly in treating severe infections caused by CRE, are still limited. To address these crucial gaps, the present study aimed to systematically review and meta-analyze existing research concerning the effectiveness and safety of CAZ/AVI in treating multidrug-resistant *Escherichia coli (E coli*) infections.

## 2. Materials and methods

### 2.1. Search strategy

Throughout the systematic review and subsequent synthesis of findings, we rigorously adhered to the Preferred Reporting Items for Systematic Reviews and Meta-Analyses framework.^[7]^ The meta-analysis was structured around the Patient, Intervention, Comparison, Outcome (PICO) framework, elucidating the following aspects: patient (P): individuals suffering from multidrug-resistant *E coli* infections. Intervention (I): Treatment involved cefotaxime and avibactam. comparison (C): The comparison was with standard treatment regimens for multidrug-resistant *E coli* infections or placebo where applicable. Outcome (O): The outcomes measured were the efficacy and safety of the CAZ/AVI combination in treating multidrug-resistant *E coli* infections, with metrics including but not limited to treatment success rate, adverse effects, and microbiological eradication.

A comprehensive literature search was performed on May 6, 2023, across 4 primary scientific databases: PubMed, Embase, Web of Science, and Cochrane Library, without temporal restrictions. The search strategy employed key terminologies like “ceftazidime avibactam,” “CAZ/AVI,” “multidrug-resistant,” “e. coli,” and “infections” to capture the extensive purview of the PICO components and to ensure an exhaustive compilation of pertinent studies. No linguistic constraints were imposed on the searches. Additionally, the reference sections of the germane articles were manually scrutinized to identify any supplementary records that may have been overlooked.

### 2.2. Inclusion criteria and exclusion criteria

For inclusion in the systematic review, studies were required to meet the following criteria: (1) clinical studies where the intervention group was treated with a therapeutic regimen that included cefotaxime and avibactam; (2) the control group was subjected to alternative treatment modalities; (3) the pathogen under study was multidrug-resistant Enterobacteriaceae bacteria; and (4) outcome metrics encompassing cure rates, bacterial eradication rates, mortality rates, and the incidence of adverse drug reactions (ADR).

The criteria for exclusion were delineated as follows: (1) studies that were duplicates or had multiple publications; (2) manuscripts presenting incomplete or ambiguous analytical data, or with outcome measures that were inconsistent; (3) studies with poor quality and lack of original data; and (4) types of articles excluded were case reports, commentaries, expert opinions, and narrative reviews.

### 2.3. Data extraction

In line with stringent meta-analytic procedures, literature screening and data extraction were conducted independently by 2 assessors and their results were cross verified for accuracy. If inconsistencies arose, the evaluators convened focused discussions to reconcile differences; if consensus remained elusive, consultation with a third impartial reviewer was initiated. The extracted data encompassed key variables, such as the first author, date of publication, study design, type of pathogen, mean age of participants, duration of follow-up, case inclusion count, therapeutic regimen, clinical cure rate, bacterial eradication rate, mortality rate, ADR, quality of evidence, and study quality score. If relevant data were absent in the published reports, the original investigators were contacted via email to request undisclosed information.

### 2.4. Quality assessment

The methodological rigor of the incorporated studies was appraised using the Cochrane Collaboration’s Risk of Bias instrument.^[8]^ Two reviewers independently examined several domains including the generation of random sequences, concealment of allocation procedures, blinding of study participants and involved staff, integrity of outcome data, selectivity in reporting outcomes, and any additional elements that could introduce bias. These dimensions were categorized as low, indeterminate, or elevated risk of bias. In cases where evaluators differed in their assessments, consensus was achieved either through deliberative dialogue or, if required, by seeking the judgment of a third reviewer.

### 2.5. Statistical analyses

To rigorously evaluate the inter-study heterogeneity, chi-square statistics were used, and the degree of heterogeneity was quantified using the I^2^ index. An I^2^ value below 50%, coupled with a corresponding *P*-value of 0.10 or greater, indicated negligible heterogeneity, warranting the use of a fixed-effects model to calculate the aggregated effect size. Conversely, an I^2^ value of 50% or above or a corresponding *P*-value <.10, signaled significant heterogeneity, thereby justifying the application of a random-effects model for the synthesis of effect sizes. Sensitivity analyses were conducted to ascertain the resilience of our findings and to identify individual studies exerting undue influence on the composite effect size. This involved the stepwise exclusion of each contributing study and the recalculating of the aggregate effect size accordingly. To scrutinize potential publication bias, the symmetry of the funnel plot was examined; a symmetrical distribution would suggest a diminished risk of skewness due to publication bias. Egger linear regression test was applied as a quantitative adjunct to further assess publication bias. All statistical analyses were two-tailed, with a *P*-value <.05 considered statistically significant. Data manipulation and analysis were performed using Stata version 17 (StataCorp, College Station, TX).

## 3. Results

### 3.1. Search results and study selection

**Figure 1.**
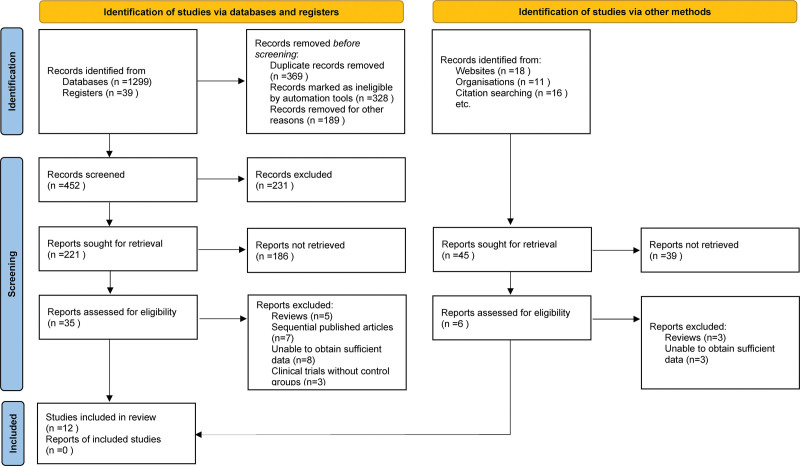
Selection process of included studies.

Upon conducting an initial search across multiple electronic databases, we identified 1383 articles that appeared to be relevant to our meta-analysis. The next phase involved meticulous removal of duplicates, which helped streamline the pool of potential articles for review. Following this, we engaged in a rigorous screening process, whereby titles and abstracts were closely examined against our predefined inclusion and exclusion criteria. This scrutiny led to a shortlisting of 35 articles that warranted further evaluation. Subsequently, these selected papers underwent a more intensive review, during which 23 were eliminated for various reasons such as inadequate data, inconsistencies in outcome measures, or failure to meet other inclusion criteria. Thus, a final cohort of 12 articles was deemed suitable for inclusion in our meta-analysis.^[9–20]^ The entire literature selection and filtration process, along with the specific rationale for exclusion at each stage, is graphically delineated in Figure 1 for comprehensive understanding and transparency.

### 3.2. Study characteristics

The studies included in this meta-analysis varied in their methodological design but were predominantly randomized controlled trials and clinical control trials. Published between 2012 and 2019, these studies predominantly focused on 2 types of pathogenic infections: carbapenem-resistant Enterobacteriaceae and Polymicrobial Infections, including multidrug-resistant Enterobacteriaceae. The average age of the participants ranged mainly between 18 and 90 years, although some studies indicated an age range above 18 years, without specifying an upper limit. The follow-up durations varied significantly across studies, with some studies not reporting this metric (NA). In terms of treatment regimens, ceftazidime/avibactam is the most commonly used, either alone or in combination with metronidazole. Sample sizes across the studies differed considerably, ranging from as few as 8 in the control group to as many as 529 in both the experimental and control groups. The evaluation metrics also vary, but most studies have assessed the Clinical Cure Rate, Bacterial Clearance, Mortality, and ADR. Despite the heterogeneity in study characteristics, the predominant focus remains on the effectiveness and safety of ceftazidime/avibactam-based treatment regimens for bacterial infections that are either carbapenem-resistant or polymicrobial, including multidrug-resistant strains (Table 1).

**Table 1:** Characteristics of studies included in the meta-analysis.

First author	Publication year	Study type	Pathogen type	Average age (years)	Follow-up duration (days)	Sample size (experimental/control)	Treatment regimen
Alraddadi	2019	Clinical Control Trial	Carbapenem-resistant Enterobacteriaceae	Above 18	NA	10/28	Ceftazidime/avibactam
Carmeli	2016	Randomized Control Trial	Polymicrobial Infection (Including multidrug-resistant Enterobacteriaceae)	18–90	28–32	154/148	Ceftazidime/avibactam
Castón	2017	Clinical Control Trial	Carbapenem-resistant Enterobacteriaceae	Above 18	NA	8/23	Ceftazidime/avibactam
King	2017	Clinical Control Trial	Carbapenem-resistant Enterobacteriaceae	18 and above	NA	33/27	Ceftazidime/avibactam
Lucasti	2013	Randomized Control Trial	Polymicrobial Infection (Including multidrug-resistant Enterobacteriaceae)	18–90	28–42	26/17	Ceftazidime/avibactam + metronidazole
Mazuski	2016	Randomized Control Trial	Polymicrobial Infection (Including multidrug-resistant Enterobacteriaceae)	18–90	42–49	529/529	Ceftazidime/avibactam + metronidazole
Qin	2017	Randomized Control Trial	Polymicrobial Infection (Including multidrug-resistant Enterobacteriaceae)	18–90	42–49	215/217	Ceftazidime/avibactam + metronidazole
Shields	2017	Clinical Control Trial	Carbapenem-resistant Enterobacteriaceae	25–91	NA	13/96	Ceftazidime/avibactam
Torres	2018	Randomized Control Trial	Polymicrobial Infection (Including multidrug-resistant Enterobacteriaceae)	18–90	28–32	405/403	Ceftazidime/avibactam
Tumbarello	2019	Clinical Control Trial	Carbapenem-resistant Enterobacteriaceae	23–88	NA	104/104	Ceftazidime/avibactam
Vazouez	2012	Randomized Control Trial	Polymicrobial Infection (Including multidrug-resistant Enterobacteriaceae)	18–90	28–42	68/67	Ceftazidime/avibactam
Wagenlehner	2016	Randomized Control Trial	Polymicrobial Infection (Including multidrug-resistant Enterobacteriaceae)	18–90	45–52	511/509	Ceftazidime/avibactam

NA: not available.

### 3.3. Results of quality assessment

**Figure 2.**
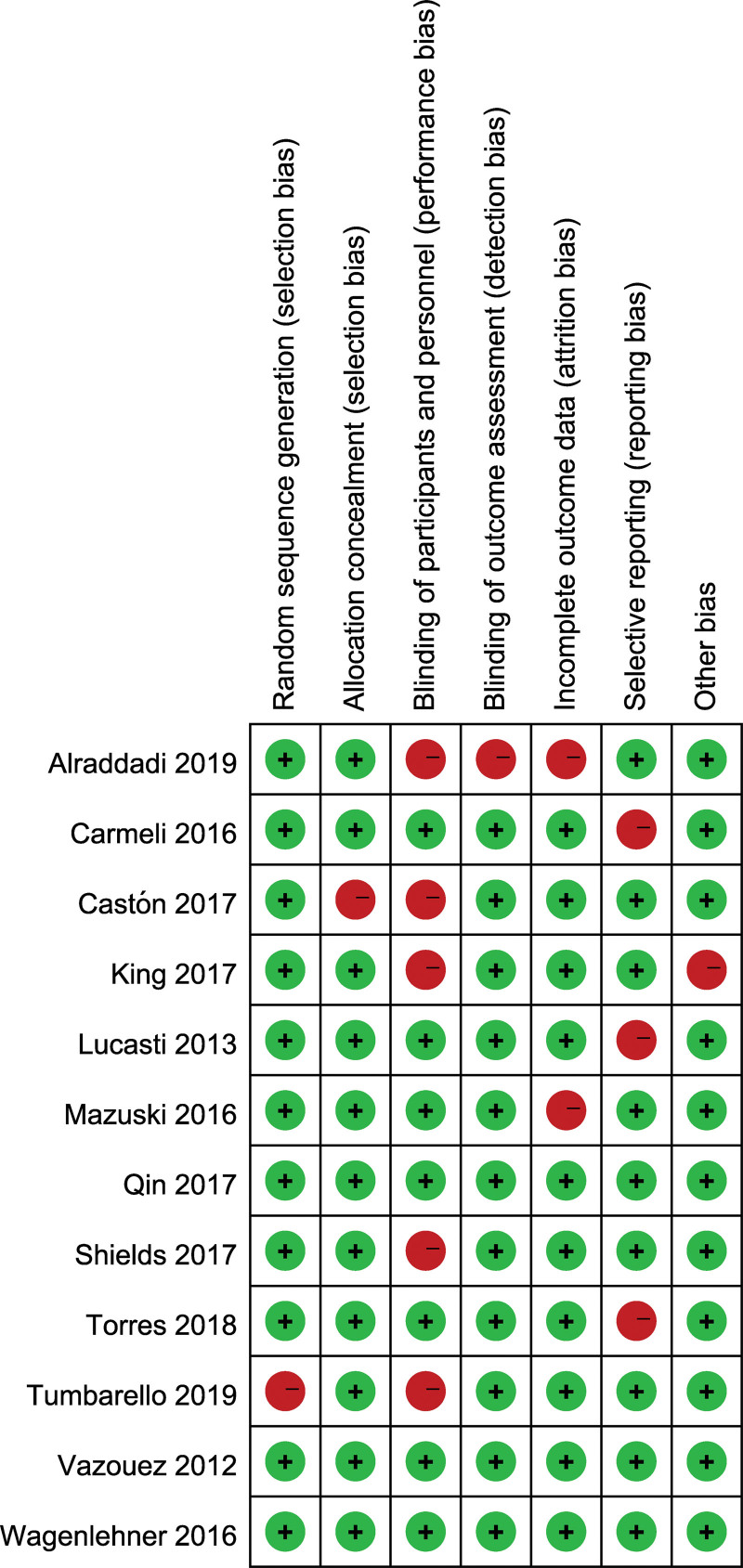
Quality assessment of included studies using Cochrane Collaboration’s tool criteria. Red in figure indicates high risk, yellow represents unclear risk and green means low risk.

The assessment of susceptibility to bias spanned diverse domains within the 12 selected studies. Of these, 3 manifested a low susceptibility to bias across all evaluated categories, signifying a robust methodological foundation. Nevertheless, 41.7% of the studies exhibited an elevated risk in the specific domain of blinding for both participants and involved personnel, thus implying the potential for performance bias to alter study outcomes. Additionally, a heightened risk of selective reporting bias was identified in one-quarter of the incorporated studies. This raise concerns that incomplete or preferential outcome documentation could have affected the comprehensive findings of these investigations (Fig. 2).

### 3.4. Meta-analysis on clinical cure rate

**Figure 3.**
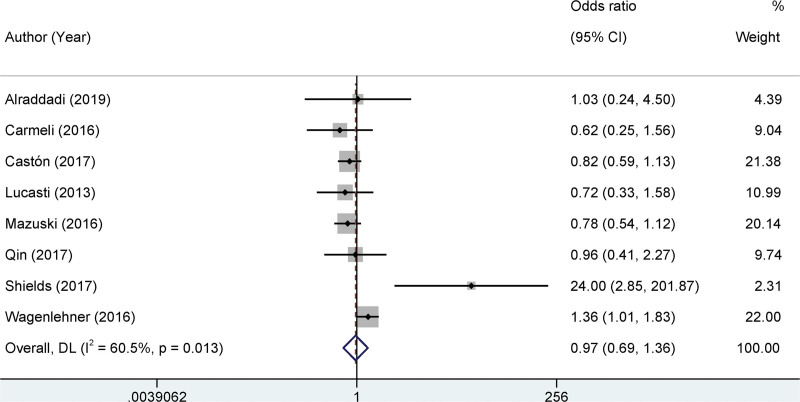
Forest plots of the clinical cure rate.

In this meta-analysis, a comprehensive review of 8 studies was conducted to evaluate the clinical cure rate. A pivotal step in any meta-analysis is the assessment of the heterogeneity among the included studies. In our analysis, significant statistical heterogeneity was identified across studies (I^2^ = 60.5%, *P* = .013). Given the presence of this heterogeneity, we used a random-effects model for our subsequent analysis to provide a more conservative estimate of the pooled effect. Our findings indicate that there was no significant difference in the clinical cure rates between the 2 groups. The odds ratio (OR) was 1.97, with a 95% confidence interval ranging from 0.69 to 1.36. Importantly, this difference was not statistically significant (*P* = .86), suggesting that the interventions or treatments compared in these studies had comparable effects on the clinical cure rates (Fig. 3).

### 3.5. Meta-analysis on bacterial clearance rate

**Figure 4.**
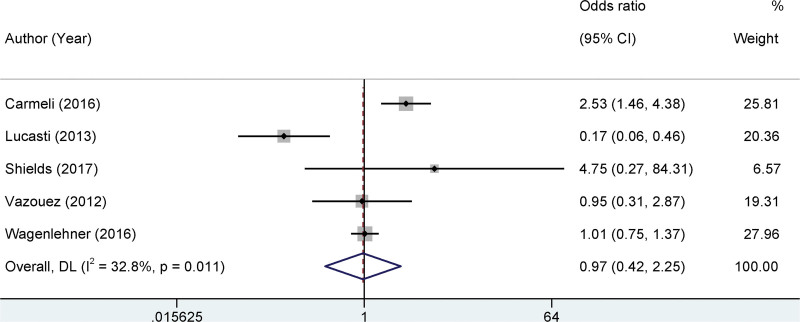
Forest plots of the bacterial clearance rate.

This section of our meta-analysis focuses on the bacterial clearance rate, which is a pivotal clinical outcome in the assessment of antimicrobial interventions. Based on our thorough literature review, a total of 5 separate studies provided data on bacterial clearance rates. Heterogeneity is the cornerstone of meta-analyses that dictates the analytical approach. Low statistical heterogeneity was detected among the studies (I^2^ = 32.8%, *P* = .011). Consequently, we employed a fixed-effects model, which assumes that all studies estimate the same underlying effect and is generally preferred in the presence of low heterogeneity. Upon pooling the data, our findings revealed that the bacterial clearance rate between the 2 evaluated groups did not exhibit a statistically significant difference. The calculated OR was 0.97, with 95% CI ranging from 0.42 to 2.25. The associated *P*-value of .36 further underscores the absence of a statistically significant difference between the groups. Therefore, it is imperative to interpret these findings in a broader clinical context. Although our analysis indicated no significant difference in bacterial clearance rates, other clinical endpoints and qualitative factors might still differentiate the interventions or treatments (Fig. 4).

### 3.6. Meta-analysis on mortality rate

**Figure 5.**
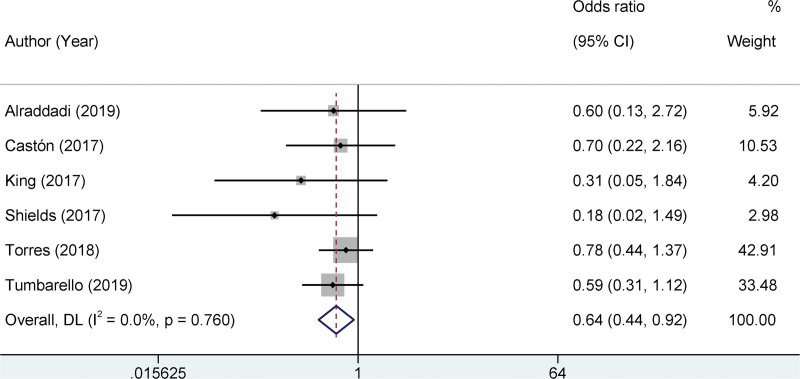
Forest plots of the mortality rate.

In clinical research, understanding the mortality rates is indispensable. The current meta-analysis revolves around this critical endpoint by drawing upon data from 6 identified studies. A key step in meta-analysis is determining the presence of heterogeneity across studies. Notably, our analysis identified no statistical heterogeneity among the included studies (I^2^ = 0%, *P* = .760). Given the absence of heterogeneity, we employed a fixed-effects model in our analysis. This model operates under the assumption that the true effect size is consistent across all incorporated studies. A significant observation emerged upon aggregating these findings. The experimental group demonstrated a significantly lower mortality rate than the control group. Specifically, the OR was computed as 0.64, with a 95% confidence interval ranging from 0.44 to 0.92. A *P*-value of .012 underscores this significant difference. These results support the efficacy of the interventions or treatments analyzed in the experimental group, as reflected by the reduced mortality rate (Fig. 5).

### 3.7. Sensitivity analysis on clinical cure rate

**Figure 6.**
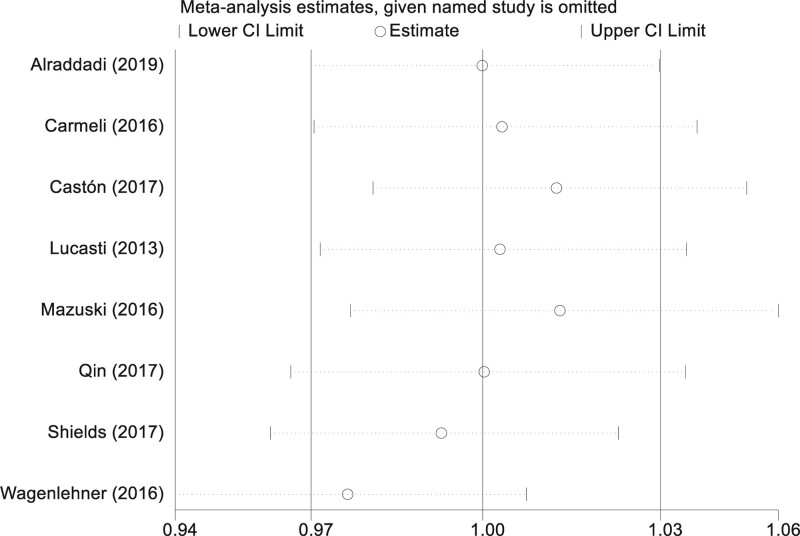
Sensitivity analysis on clinical cure rate.

Amidst the discernible heterogeneity exhibited by the studies incorporated in our meta-analysis, we undertook a meticulous sensitivity examination to gauge the steadfastness and trustworthiness of the aggregated outcomes. Through a systematic approach, we excluded studies sequentially, recalculating the composite effect measures with the residual studies each time. This comprehensive exercise affirmed that the aggregated outcomes consistently held their ground, showcasing unwavering stability, regardless of the omission of any single investigation. This observation fortifies the notion that no singular study held a disproportionate sway over the collective results, amplifying the dependability of our derived conclusions. Resilience manifested by the outcomes across the gamut of these analyses buttresses the veracity of our principal determinations, lending further credence to the inferences of this meta-analysis (Fig. 6).

### 3.8. Assessment of publication bias in meta-analysis

**Figure 7.**
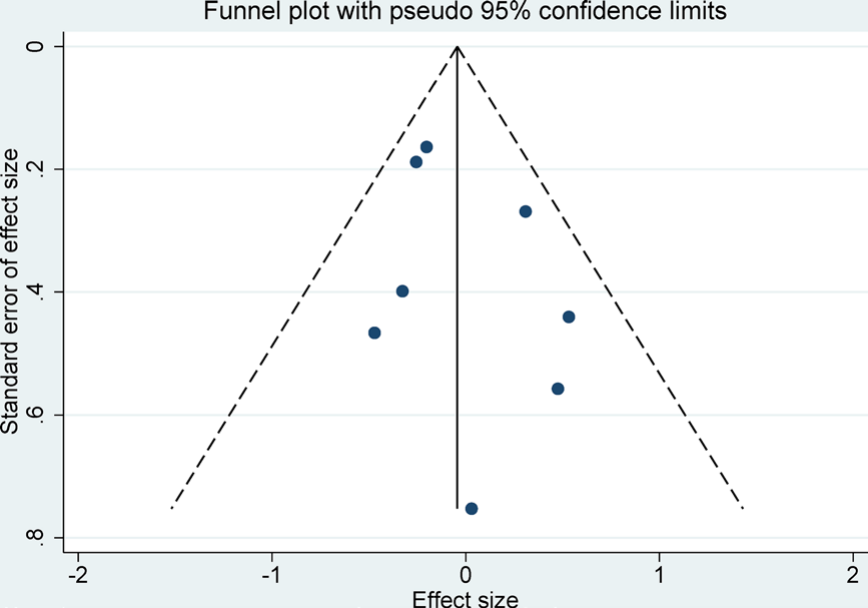
Funnel plot for publication bias in all included studies.

Funnel plots, derived from the studies included in our meta-analysis, demonstrated symmetrical patterns, signifying the absence of substantial publication bias, as shown in Figure 7. Validation using Egger linear regression revealed no detectable publication bias across the varying parameters (*P* > .05). These findings bolster the integrity and stability of the outcomes of this meta-analysis.

## 4. Discussion

The onset of MDR-EB infections has increased prominently, manifesting as a formidable global health dilemma. These infections, characterized by their tenacity against frequently prescribed antibiotics, have catalyzed increased rates of morbidity and mortality along with extended hospitalization durations. The collective consequence is an amplifying strain on global healthcare infrastructures.^[21,22]^ The innovative therapeutic advent of CAZ/AVI is central to this medical quandary. This combination, marrying the strengths of a third-generation cephalosporin with a non-beta-lactam beta-lactamase inhibitor, represents an advanced frontier in the battle against MDR-EB infections.^[23]^ Its potential benefits, especially in scenarios where other therapeutic interventions have been faltered. The breadth of clinical research probing its effectiveness, mechanisms of bacterial elimination, and comprehensive safety parameters has set the stage for its prospective mainstream therapeutic inclusion.

Underscoring this clinical landscape, our meta-analysis assumed critical relevance. By meticulously amalgamating an array of research studies focusing on the prowess of CAZ/AVI against MDR-EB infections, we strived to deliver a holistic understanding of its therapeutic spectrum. Diving into the specifics, our meta-analysis juxtaposed antimicrobial regimens containing CAZ/AVI against those devoid of it, aiming to discern the relative effectiveness and safety in treating MDR-EB infections. In terms of efficacy, our analysis revealed a nuanced picture. While both treatment groups—those incorporating CAZ/AVI and those excluding it—exhibited comparable clinical cure rates and bacterial eradication metrics, the former distinctly marked a significant reduction in patient mortality rates.

This pattern may be attributed to multiple intertwined factors. The emergence of drug-resistant strains coupled with the diminished inhibitory efficiency of traditional beta-lactam inhibitors against carbapenem compounds has necessitated the evolution of “second-generation” beta-lactamase inhibitors. This category, which prominently includes AVI, hinges on a non-beta-lactam structure, broadening its antimicrobial spectrum to counteract A-class carbapenemase-producing CRE infections such as those caused by Klebsiella pneumoniae. Nevertheless, the sheer complexity of MDR-EB infections, characterized by severe clinical presentations, extended disease trajectories, and a confluence of concurrent medical conditions, may mitigate enhancements in clinical cure rates and bacterial clearance. Furthermore, the pharmacological attributes of CAZ/AVI deserve special mention. It has minimal potential for drug-drug interactions and favorably influences the patient tolerability index.^[24,25]^ In addition, its administration is rarely associated with severe ADRs. This suggests that when employed against MDR-EB infections, CAZ/AVI could substantially mitigate fatalities emanating not from the primary infection per se but from drug-induced adversities. Consequently, its preferential positioning in treating grave infections, especially those caused by CRE pathogens, has emerged as an invaluable therapeutic alternative, potentially revolutionizing treatment paradigms.^[26,27]^

In our meta-analysis, heterogeneity was discernible, potentially stemming from multiple determinants. First, the diversity in pathogen types is salient; whether the infections were caused by a singular strain or multiple strains could significantly impact outcomes. Additionally, the caliber of the cited literature might introduce variability; high-quality studies often employ rigorous methodologies, yielding potentially different results than studies with lower rigor. Moreover, the therapeutic approach used in the test groups introduced another layer of complexity. Combining medications or the presence (or absence) of follow-up could profoundly influence the bacterial clearance rates. Taken together, these factors underscore the necessity of caution when interpreting and generalizing our meta-analysis results.

Our study demonstrates that the cefotaxime–avibactam combination is an effective and safe alternative for treating multidrug-resistant E coli infections, particularly where traditional antibiotics are ineffective. This aligns with personalized medicine approaches and suggests a need for policy reforms to integrate such treatments into standard care, improving outcomes in areas with high antibiotic resistance. The findings encourage pharmaceutical research towards novel β-lactam/β-lactamase inhibitor combinations, addressing antibiotic resistance concerns. Further large-scale trials are needed to validate these results and assess long-term impacts on resistance patterns and health outcomes. Our research offers crucial insights for clinical and pharmaceutical advancements in managing multidrug-resistant infections.

Our study had several limitations that warrant Acknowledgments. First, the diversity of the included studies, each with its own methodology, introduces potential inconsistencies in data collection and analysis. This variability could have affected the pooled results and generalizability. Additionally, the heterogeneity observed in certain outcomes, although addressed through statistical methods, might still mask nuances related to specific patient populations or treatment protocols. Thus, it is essential to interpret our findings in the context of these constraints.

## 5. Conclusions

In conclusion, CAZ/AVI demonstrated a significant reduction in patient mortality, positioning it as a pivotal therapeutic alternative for MDR-EB infections, especially in cases of CRE. Although our findings have substantial clinical implications, it remains imperative to further corroborate these conclusions through well-structured, large-scale clinical trials.

## Author contributions

**Conceptualization:** Geming Liu.

**Formal analysis:** Geming Liu.

**Methodology:** Jia Qiu, Zhisen Liu.

**Resources:** Jia Qiu, Yang Liu, Zhisen Liu.

**Software:** Jia Qiu, Yang Liu, Zhisen Liu.

**Supervision:** Geming Liu.

**Validation:** Yang Liu.

**Writing – original draft:** Geming Liu.

**Writing – review & editing:** Geming Liu.
